# Internet of Measurement Things Architecture: Proof of Concept with Scope of Accreditation †

**DOI:** 10.3390/s20020503

**Published:** 2020-01-16

**Authors:** M. Cagri Kaya, Mahdi Saeedi Nikoo, Michael L. Schwartz, Halit Oguztuzun

**Affiliations:** 1 Department of Computer Engineering, Middle East Technical University, 06800 Ankara, Turkey; oguztuzn@ceng.metu.edu.tr; 2 Mathematics and Computer Science, Eindhoven University of Technology, 5600 Eindhoven, The Netherlands; m.saeedi.nikoo@tue.nl; 3 Cal Lab Solutions, P.O. Box 111113, Aurora, CO 80042, USA; mschwartz@callabsolutions.com

**Keywords:** calibration laboratory, industrial internet of things, internet of measurement things, metrology information infrastructure, measurement uncertainty, scope of accreditation

## Abstract

Many industries, such as manufacturing, aviation, and power generation, employ sensitive measurement devices to be calibrated by certified experts. The diversity and sophistication of measurement devices and their calibration needs require networked and automated solutions. Internet of Measurement Things (IoMT) is an architectural framework that is based on the Industrial Internet of Things for the calibration industry. This architecture involves a layered model with a cloud-centric middle layer. In this article, the realization of this conceptual architecture is described. The applicability of the IoMT architecture in the calibration industry is shown through an editor application for Scope of Accreditation. The cloud side of the implementation is deployed to Microsoft Azure. The editor itself is created as a cloud service, and IoT Hub is used to collect data from calibration laboratories. By adapting the IoMT architecture to a commonly used cloud platform, considerable progress is achieved to encompass Metrology data and serve the majority of the stakeholders.

## 1. Introduction

The usage of the Internet of Things (IoT) spreads to different domains as it provides opportunities in big data analytics, machine learning, and cloud computing technologies in the industry. This emerging approach is called the Industrial Internet of Things (IIoT) or Industry 4.0. Considering the benefits of this new trend, such as increased productivity, short development periods, quickly developed customized products, and resource efficiency [1], numerous solutions are proposed in different areas, including farming, manufacturing, and telecommunications. When these characteristics of IIoT technologies are taken into account, they seem to provide promising solutions for the open research problems in Metrology and the calibration industry.

The notion of the “Internet of Measurement Things (IoMT)” was proposed in [2] as a layered IIoT architecture that separates physical equipment, cloud-based services, and applications. This architecture is inspired by the Metrology Information Infrastructure (MII) initiative and previous experiences with the Metrology.NET platform. MII and Metrology.NET are efforts to develop community-driven standards and to increase the usage of automation in the Metrology world. IoMT architecture aims to advance previous work into an IIoT-based solution.

The IoMT architecture has three layers, namely *physical*, *MII Cloud Services*, and *application*. The physical layer contains equipment for calibration, generally in calibration laboratories (CLs). The MII-Cloud Services layer hosts the services that are provided for the calibration industry. The application layer constitutes different sorts of software that can be used in Metrology and the calibration industry, e.g., calibration automation systems, asset tracking systems, and scope of accreditation (SoA) editors.

CLs operate with different capabilities. Their services may cover one or more disciplines such as Dimensional, Electrical, and Mechanical. Customers choose CLs by checking their capabilities and whether the lab is certified by accreditation bodies (ABs) pertaining to these capabilities. However, using current methods, customers often have limited options to find the lab that best suits their needs.

An accreditation certificate declares the lab’s capabilities and guarantees an approved quality of service to customers. For an accredited CL, SoA represents a documented list of calibration fields, specific measurements, uncertainty values, and other parameters. A certificate of accreditation is accompanied by the scope document, and the certificate is incomplete without it. The SoA document includes only the calibration areas that a laboratory is accredited for, and only the listed areas may be offered as accredited calibrations to customers. The format and other details are usually defined by the AB.

In this article, the realization of the conceptual IoMT architecture is elaborated as an extended version of the work presented in [3]. An SoA editor is chosen for the case study. Previous work showed the principles of the editor, some implementation detail, and how the editor fits the IoMT architecture. However, the relationship of the editor with the cloud environment was conceptually explained. Specific to this article, one of the well-known and well-established cloud computing service providers, Microsoft Azure, is employed as the underlying platform, and the SoA editor is implemented as a cloud service. The data flow from the calibration devices in the labs to the cloud is managed by Azure IoT Hub.

Previous work of the IoMT concept and contribution of this paper are summarized as follows:

- **Previous work-1:** The IoMT architectural framework was presented in [2]. Based on MII concepts and Metrology.NET experiences, an IIoT-based architecture was conceptually introduced to the calibration community.
- **Previous work-2:** The SoA editor was introduced in [3] as an application conforming to the previously proposed IoMT architecture. The components of the SoA editor fit the architecture. However, the connections among the components of the editor and the MII services remained conceptual.
- **In this article;**
  - The realization of the IoMT architecture is explained, including cloud-side implementations.
  - Microsoft Azure is chosen as the cloud provider.
  - The SoA editor presented in [3] is chosen for the case study and re-implemented as a cloud service.
  - The implementation is partial for proof of concept. On the other hand, the necessary steps to implement a full-scale IIoT application using Azure are explained.
  - Calibration devices are employed as IoT devices, and Azure IoT Hub is used to collect data from them.

Although some research enriches Metrology with IoT technologies in recent years, we can still say that we are witnessing the beginning of this transition process. Moreover, to the best of our knowledge, there is not much work done to make calibration automation easy for all stakeholders in the industry. In these aspects, this work is a contribution to adapt Metrology and the calibration industry to IoT technologies in the industrial scale. This improvement would ease the development of new applications that use machine learning and big data analytics.

We employ calibration equipment as data producers of an IoT application. In this sense, sensors in a common IoT application are replaced with calibration equipment in the IoMT architecture. From another viewpoint, our architecture comprises applications and labs that focus on sensor calibration. An example of the use of IoT in sensor calibration is provided in [4]. Moreover, standardization is essential for sensor calibration [5]. Using the cloud for commonly used services would help compliance with standards.

The rest of the article includes required background information that covers the SoA concept, the MII initiative, the Metrology.NET platform, and Microsoft Azure along with its IoT-related services. The related work is discussed, including similar IIoT applications from different industries and similar work that use Azure. After the related work section, the IoMT architecture is elaborated. Then, the SoA editor is provided in detail along with the cloud implementation: how to develop the SoA editor as a cloud service and other Azure services used in the proof of concept case study. The article is concluded with remarks and possible future work.

## 2. Background

In this section, an introduction to the essential calibration domain terminology used in this work is provided. A brief overview of the MII is also given.

### 2.1. Calibration

Calibration is an area of metrology, the science of measurement. It indicates the quality and accuracy of measurements performed in a domain. Measurement equipment may have errors in their results as time progresses. These drifts can be caused by misuse or some external factors, such as temperature and humidity.

Each measurement device has a margin of error on its measurements, namely the measurement uncertainty. By adjusting the device, it is intended to minimize the measurement uncertainty or ensure that it remains at an acceptable level. Adjustment is a different step that is separate from calibration and usually follows a calibration job. Calibration only includes the testing of a customer unit, while the adjustment is made to fix the possible divergence of a unit from its regular operation [6].

### 2.2. Scope of Accreditation

All CLs that are accredited as per ISO/IEC 17025 [7] (International Organization for Standardization/International Electrotechnical Commission) have an SoA. The scope of a lab represents its technical capabilities for which the lab has requested the accreditation and was deemed to be competent by an AB. Customers can refer to the SoA of a CL to find out if their unit can be calibrated by that lab and with how much accuracy. The scope includes further details such as testing methods, types of inspections, and certifications. This way, customers can also compare calibration service providers and choose the one that best suits their needs.

Calibration and Measurement Capability (CMC) represents the best achievable measurement uncertainty of ideal measurement equipment under normal operational conditions in a CL. ABs assess CLs by these numbers, based on CLs’ personnel, equipment, and processes. These values are represented in the form of constant values or as formulas.

Table 1 shows an excerpt extracted from the SoA certificate of a CL issued by an AB. The excerpt includes two parameters concerning two different calibration disciplines covered in the CL scope. Temperature-Measure (first row) is a Thermodynamics parameter, and DC Voltage-Measure (second row) represents an Electrical parameter. The table shows uncertainty values (CMC column) for the specified ranges using specific test equipment mentioned in the *Comments* column. The *Range* column may include a fixed point or a range of values. Similarly, the *CMC* column may include a constant value or a CMC equation (second row). The parameters and the formulation shown in an SoA certificate may consist of other variations and more complexities than what is shown in Table 1.

Particular infrastructure needs to exist to provide better solutions for SoA related problems. Such an infrastructure would provide data type standardization, communication protocols, technologies, and services, and it would lay the foundation for automation solutions for accreditation processes. In recent years, there have been endeavors in the advancement of such an infrastructure. The NCSLI (National Conference of Standards Laboratories—International) MII working group is one of the most active metrology communities working on digitalizing the SoA activities [8].

### 2.3. Metrology Information Infrastructure

Standards are essential for metrology to achieve reliable results. Similar to other domains, metrology also needs automation and standardization. Standards can help to increase quality in metrology when applied to various processes or data. Examples can be listed as documentation, conformance testing, risk analysis, uncertainty analysis, product inspections, service procurement, and accreditation.

Mark Kuster introduced MII in NCSLI Metrologist magazine in 2013 [9], initially as Measurement Information Infrastructure. The motivation of MII is standardizing data types in metrology, including SoA, instrument specification sheets, and calibration and testing certificates [10]. In this way, it is aimed to ease the communication of world-wide measurement-related systems and replace manually processed documents with unambiguous machine-readable ones.

**Figure 1:**
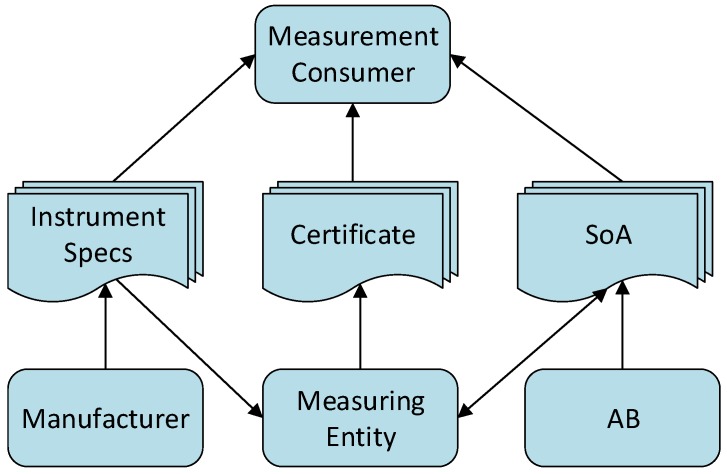
A Partial view of the metrology information flow.

Figure 1 illustrates the flow of data among some critical stakeholders of the metrology domain. Instrument specs, certificates, and SoA are different types of metrology data shared among the stakeholders. Arrows in the figure represent the directions of the data flow.

### 2.4. Metrology.NET

Metrology.NET is a distributed automation platform for calibrating test equipment [11,12]. The platform design follows a modular approach for data management and calibration automation. The platform connects multiple sub-systems to form a system of systems to fill in the gaps among various metrology software systems currently used at CLs. It follows the Lego^®^ analogy, where sub-systems are similar to Legos that connect using a connector layer. The standardized connector layer is supposed to join different systems together, which allows the user to build up a total solution by choosing and configuring smaller block systems to work together.

**Figure 2:**
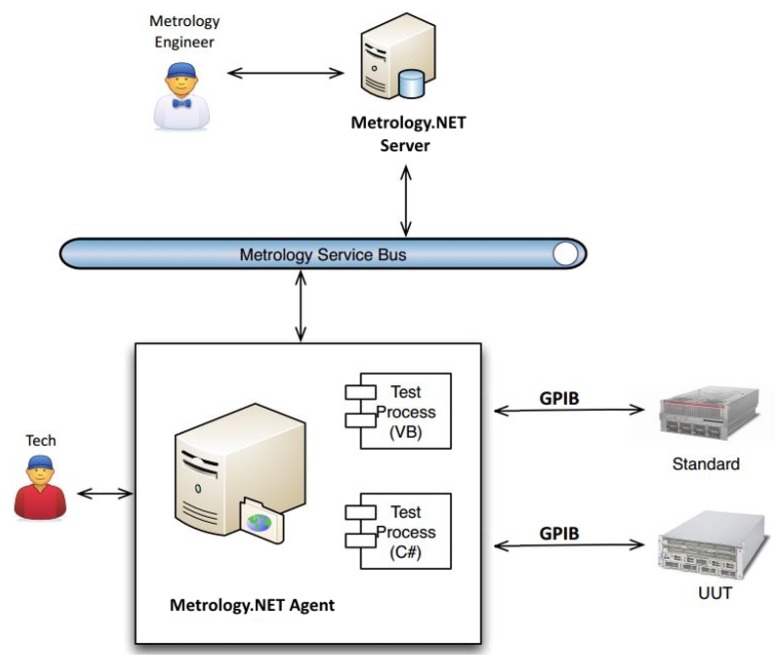
An overview of the Metrology.NET system.

Figure 2 shows an overview of the Metrology.NET platform, and the system decomposition with the connections among system components. The metrology engineer is usually in charge of the server-side that commands the agents for their jobs. The agent (testing terminal) is the computing machinery that communicates directly and physically with the testing hardware, including the unit under test (UUT) and reference equipment. The agent runs the automation software. The typical way of connecting the agent to the UUT and reference equipment is through the GPIB (General Purpose Interface Bus).

The Metrology.NET platform follows a client-server architectural style. The server side hosts the application services, and the clients act as testing workstations that run the automation. Depending on a CL capacity, it can only consist of a single machine running both the server and the client, or there may be a central server machine with several client terminals that consume the services provided by the server. The web interface of the server-side allows technicians who run calibration work orders to locally or remotely control and interact with the running automation process.

The server side of the platform can be physically located anywhere, as long as there is a network connection between the server and the client(s). A secure and fast network connection between the remote server and the clients ensures automation gets done in the correct order. The clients act like worker bees that collaboratively do the calibration job. They are all controlled through the central server. The server side also keeps a database of the calibration-related data, including test points. This provides a centralized monitoring of the increasing data and the involved processes. The server can manage shared calibration jobs that can occur across labs and the data communication among labs. Another option for the server is to be hosted by a cloud service and take advantage of the cloud technologies if that helps the business.

Metrology.NET seeks for separation of concerns in handling calibration data and processes. A calibration technician examines a calibration work order in terms of a set of test points for specific customer equipment. From that aspect, the calibration task is the process of collecting measurement results for these test points. Once the results are obtained, the job is almost complete, and the server-side can review these data and issue the related certifications for the tested instrument.

Fully automated calibration increases productivity, accuracy, and repeatability in calibration processes. In Metrology.NET, a calibration task is a composition of smaller reusable test modules that aim to test specific functionalities of a UUT using known reference equipment configuration. Multiple subsets of test points may be passed to different modules to perform the automation. Modules are loosely coupled, and they handle their own job and send their results back to the application server.

### 2.5. Microsoft Azure and IoT Services

Microsoft Azure is a cloud computing platform that contains Infrastructure as a Service (IaaS), Platform as a Service (PaaS), and Software as a Service (SaaS). Providing flexibility in using different programming languages and communication protocols, Azure simplifies the development, deployment, and management of distributed applications for various application fields.

Azure Cloud Services is the PaaS environment of Azure [13]. Locally created applications are deployed to Azure cloud service and can run together with other Azure services to compose a broader application.

Microsoft provides a set of cloud services to connect, monitor, and control IoT assets [14]. These services are collectively called *Azure Internet of Things*. Some of these services are *IoT Central*, *IoT solution accelerators*, *IoT Hub*, and *Azure Digital Twins*. Users can select the service they need by deciding the amount of control they want to have over the application. Also, some of these services provide templates based on common IoT solutions. Alternatively, users can build their applications from scratch.

## 3. Related Work

This section contains related work in two main categories: Metrology and IoT applications, and Azure usage in the contexts of IoT and IIoT.

### 3.1. Metrology and IoT

The history of using IoT technologies in metrology applications is not very old. In one of the recent works, Lazzari et al. [15] discuss the *smart metrology* term, arguing that the big data collected in the industry is meaningful when it is reliable. Smart metrology, a new interpretation of metrology based on reliability, is presented as a solution. As an example of the benefits of this approach, it encourages Metrologists to re-evaluate calibration intervals instead of regular calibrations enforced by the law.

In a literature survey, Daponte et al. present measurement applications based on IoT [16]. The authors investigate related work in different application fields: intelligent transportation systems, smart and connected health, smart energy, smart environment, smart building, and smart factory.

In a white paper [17], Monnier presents smart grid solutions and combines smart meters with IoT. Also, existing smart grid connection approaches in the literature are discussed. Then, solutions provided by the author’s company for smarter and more connected smart grids are given. Angrisani et al. propose a LabVIEW-based platform for remote programming of automatic test equipment [18]. It may be hard to have all the necessary devices in the same lab when training technicians. In such a scenario, it is critical that a lab with the necessary devices shares its resources with other labs. Moreover, the platform allows connecting to a device and programming it remotely.

### 3.2. Azure usage in IoT and IIoT systems

There is plenty of research leveraging Azure in IIoT and IoT applications in the literature. This section covers some of them.

A recent study that employes Microsoft Azure in the IIoT context is conducted by Haskamp et al. [19]. They explain the process of retrofitting a legacy automation system to obtain an Industry 4.0-compliant system. In another study, Raju and Shenoy explain the benefits of using the capabilities of cloud and IoT in the industrial domain [20]. They use Azure IoT Hub in their case study.

Forsström and Jennehag evaluate an IIoT system’s monetary cost and network response time performance [21]. The system uses OPC-UA (Open Plant Communication Universal Architecture) and Microsoft Azure IoT Hub. Their case study includes a 1500-sensor real-life industrial system. They present results for fiber-based and mobile internet communication. Also, a cost-wise evaluation is provided based on the price plan of Azure IoT Hub.

Another category of research covers the Azure usage in IoT applications, not necessarily in the industrial context. Shi et al. present their robust end-to-end security solutions for IoT systems with limited budget in [22]. They integrate Azure Sphere microcontroller unit and Microsoft Azure cloud services in their solution. Azure IoT Hub is used as the cloud gateway that handles message traffic between Azure services and IoT end devices. Detailed descriptions of the system both for hardware and software components and test results are provided in the study.

Microsoft Azure IoT cloud server is used to handle real-time traffic flow-data in another study [23]. Collected data is analyzed to evaluate signal timings, and waiting times are reduced. Another work suggests controlling of smart switches through a web application and cloud technologies [24]. Microsoft Azure SQL (Structured Query Language) database is used on the cloud side. Moreover, a reference architecture is presented for IoT applications to ensure security and privacy [25]. Microsoft Azure is used as the provider in their movie suggestion application case study.

Al-Masri et al. propose an approach to improve urban waste management [26]. Recycle.io, an IoT-enabled approach, allows categorizing wastes at the time of disposal. Smart recycle bins equipped with cameras and sensors are used. Collected images and data are processed at the edge of a network and cloud. Microsoft Azure IoT Hub is used for device management.

Another similar research that employs cloud technologies in IoT applications is conducted by Ferrández-Pastor et al. [27]. The user-centered design model is used to obtain knowledge of farmers to develop IoT systems for agriculture. Edge and fog computing paradigms are used to implement IoT architecture, operating rules, and smart processes.

## 4. Internet of Measurement Things

**Figure 3:**
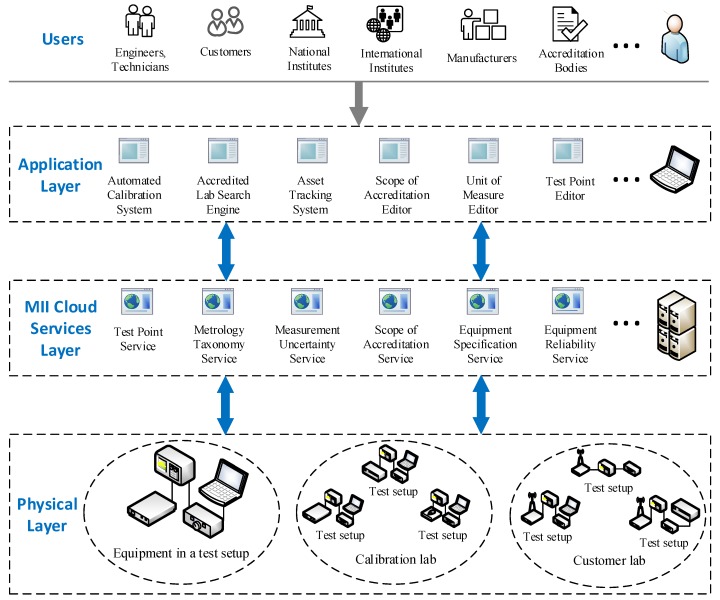
The IoMT architecture description.

This section explains the previously proposed IoMT concept and provides a detailed description of the proposed architecture. Based on the need for standardized software in the calibration industry, an architectural framework was proposed [2]. This architecture contains three layers, as shown in Figure 3: the Physical layer, the MII Cloud Services layer, and the Application layer. This layered model conforms to the reference architecture for IIoT proposed by Industrial Internet Consortium (IIC) [28].

The physical layer consists of all the physical infrastructure in the calibration industry, which produces measurement data. Physical equipment in this layer can be thought of in different scales and configurations. For example, a small setup may contain a UUT, and a reference device and a complicated setup may comprise tens of calibration setups running at the same time. The physical layer can encapsulate all organizations and domain people as a connected network at the largest scale.

The MII cloud services layer comprises the formatted measurement data and the services that use it. Robust services based on agreed standards and protocols can encourage different organizations in the domain to adhere to the standardization. Therefore, smaller solutions to the industry’s common problems are provided in this layer to be added up to create bigger solutions. To serve diverse applications, the data used by these services should be based on the standard formats and schemas developed by the MII community. In this scenario, applications that reside in the application layer should also conform to the same standards.

The application layer consists of all different sorts of software that can be exploited in the domain. Applications in this layer use services and the data stored in the MII Cloud Services layer. An application can benefit from several services, and multiple applications can use a service at the same time. Some examples of applications in this layer are automated calibration system, accredited lab search engine, unit of measure editor, and scope of accreditation editor. Some of these applications are already implemented and in use (e.g., Qualer search engine [29]); they do not have an IoT perspective, though. However, they can easily be adapted to the IoMT architecture if they use MII cloud services since they are MII-aware.

In Figure 3, different users of the calibration industry who have different connections to the domain are depicted above the application layer. Some of these users are ABs, national and international metrology institutes, CLs, calibration software companies, equipment manufacturers, and customers, namely equipment end users. The gray arrow from these users to the Application layer in the figure indicates a *uses* relationship in the UML (Unified Modeling Language) terminology. Users have access to the MII services in the MII Cloud Services layer through the applications in the Application layer.

## 5. Case Study: The SoA Editor

**Figure 4:**
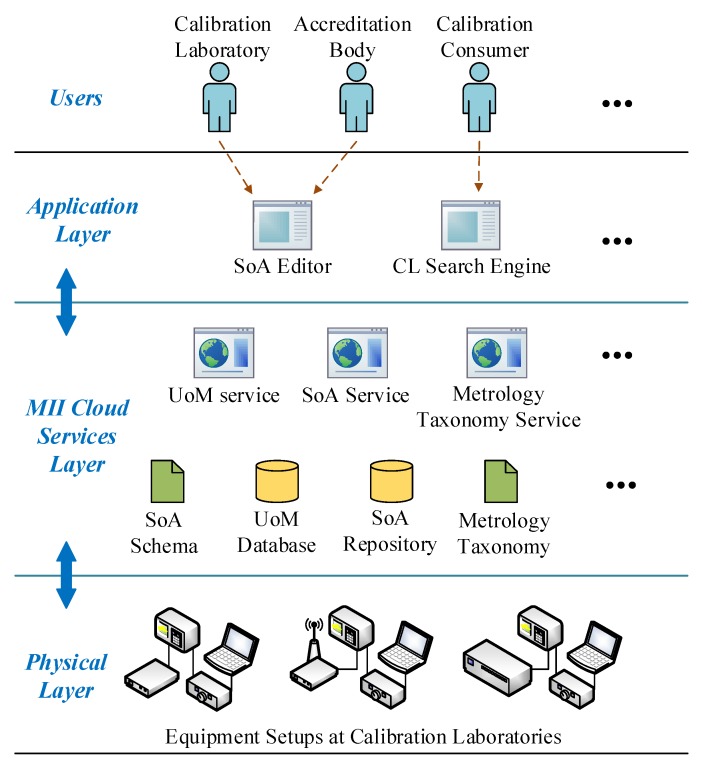
The SoA editor and the IoMT architecture.

This section explains the SoA editor and its components, also, how they all fit into the IoMT architecture. Figure 4 illustrates the SoA editor components on different layers. Conceptually, the editor sits in the Application layer, and it uses services and data on the MII-Cloud services layer. Arrows indicate this relationship in Figure 4.

The SoA editor is supposed to be used by different users of SoA data. These are specifically CLs and ABs that can interchange SoA-related data through the infrastructure provided by MII components and the medium supplied by the editor. Since the data in the cloud are stored based on specific formats and standards, third-party application developers can develop other applications conforming to the provided interfaces. An example application that uses SoA data in MII format is the Qualer Search Engine [29] that is shown as *CL Search Engine* in the Application layer in Figure 4. The engine aims to provide search capability for accredited CLs around the world. The engine works on the SoA repository developed by the MII group and for the time being works for the calibration entities in North America. The tool provides search criteria based on several parameters such as location, lab capability, and measured quantities. In the rest of the section, we will explain the traditional accreditation scenario and an alternative scenario that uses the SoA editor. Also, the details about the main MII software elements that give power to the SoA editor are explained.

### 5.1. Traditional vs. MII-Aware Calibration Laboratory Accreditation

**Figure 5:**
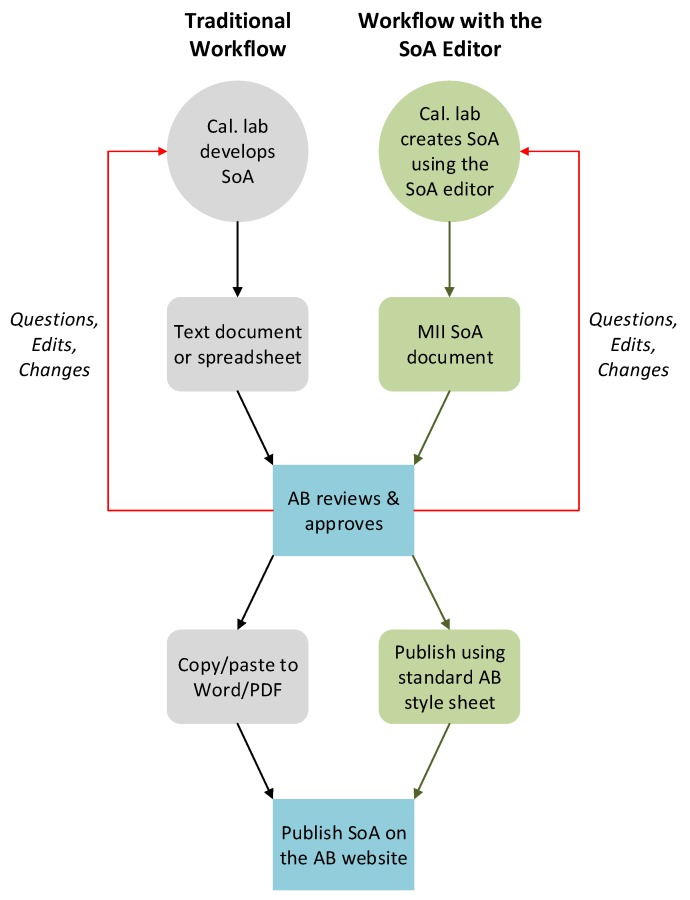
Traditional accreditation workflow vs. workflow with the SoA editor.

In this section, we present two scenarios for the accreditation process: The traditional scenario, and an MII-aware scenario. Figure 5 illustrates these scenarios. We first consider the traditional workflow of accreditation for CLs. The CL first prepares documents representing SoA based on its calibration parameters and uncertainties. These documents are then passed to an AB for assessment. The AB checks the scope, and after a detailed review, makes the decision. If it is approved, the lab scope is published on the AB’s website in a known data format. The accreditation process is composed of a detailed and complex set of activities and interactions between the CL and the AB. It involves an intensive exchange of questions, answers, and modifications to the scope. Customers can access a CL SoA through the documents published on the AB website. The left-hand side of Figure 5 summarizes the process.

We now consider an alternative scenario for the accreditation process using MII technologies. CL creates SoA using an editor that stores the data in a standard format. The tool then exports an SoA document conforming to the AB requirements. Then, the document is passed to the AB over the Internet. Similar to the traditional scenario, the AB performs the review and makes a decision. If the AB approves the scope, it publishes the scope using a standard AB style sheet on its website. Along the process of accreditation, data between the CL and the AB goes back and forth in a standard way using the SoA editor. Because of the data format standardization, other tools can be developed to use SoA data and provide several features to calibration customers. The right-hand side of Figure 5 summarizes the process.

### 5.2. SoA Schema

SoA documents are written for human readers, and typically they are not in a machine-readable format. To read these documents and interpret the information contained in the CMC in them, one needs to have in-depth technical knowledge in Metrology. An SoA data format was introduced by David Zajac [30] in 2016. In this pioneering work, he presents an XML (Extensible Markup Language) schema that helps flexibly formulate every SoA parameter into the schema. He described his initial ideas on how the schema should be designed and presented its overall functionality. An open-source API (Application Programming Interface) was also developed that allows for SoA data manipulation and calculations based on the given schema.

### 5.3. SoA Repository

The MII group is developing a cloud-hosted repository for SoA data [31]. The repository allows for validation, access, and storing data based on the presented SoA schema at [30] and other standards. Accordingly, the repository provides services offered by the MII community, such as units of measure (UoM) and metrology taxonomy. The repository presently includes several hundred thousands of CMCs from hundreds of calibration entities in the database. The data are mainly coming from North American accredited labs.

### 5.4. Units of Measure Database

A UoM database is being developed by the MII group. The aim is to provide the metrology application developers with a service that gives access to UoM data in a uniform way. An editor for the database was also developed to allow the metrology community to edit and expand the database [32]. The UoM editor takes advantage of the MathML (Mathematical Markup Language) [33] to provide a rendered presentation for each UoM.

One reason for developing such a uniform database is to resolve possible ambiguities. An example of ambiguity for UoM is the *fpm* case which can be interpreted in two ways: *Feet Per Minute* or *Flashes Per Minute*. MII presents the measurement quantity notion, which is paired with all measurement values and allows for the definition of UoMs in an unambiguous way. In this example, we would have *flash-rate* and *speed* quantities. The MII UoM also defines a *singular base UoM*, *convertable to UoM alternatives* and aliases for all UoMs. For the mentioned example, the singular base units would be *flashes-per-second* and *meters-per-second*, the convertable-to alternatives would be *flashes-per-minute* and *feet-per-minute* respectively and aliases could be *fpm* for both, since the quantities are different already.

### 5.5. Measurement Taxonomy

The measurement taxonomy aims to provide a set of naming conventions and a hierarchical data structure to encompass all types of measurements that can be taken. The MII group wants to specify unique types that can clearly distinguish every sort of measurement. The taxonomy of measurements helps to index, catalog, and easily share measurement related data. Based on the proposed convention, a measurement type starts with *source* or *measure* and progresses from general to more specific sub-categories. The taxonomy definition also includes extra information about measurement types, such as the specific required and optional input parameters used in that measurement.

**Figure 6:**
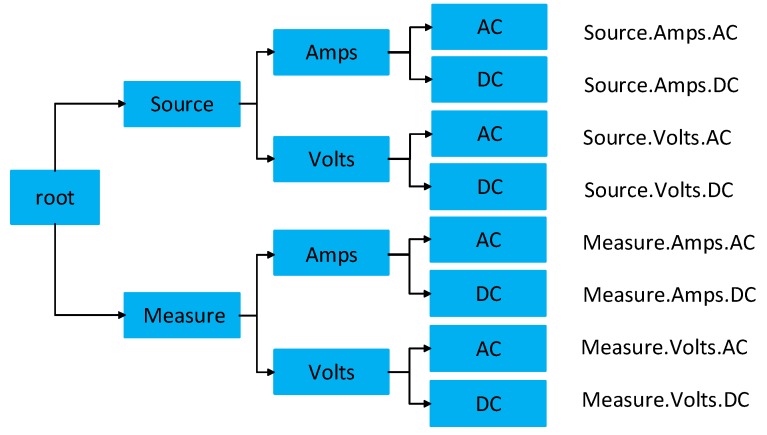
An example measurement taxonomy.

Figure 6 shows an example of measurement taxonomy in a tree view. Starting from the root, it goes down to the leaves from general to more specific categories. For example, if the measurement is about the AC voltage, the measurement type can be formulated as “Measure.Volts.AC”. The number of sub-categories for the measurement can vary based on its type. Each leaf on the taxonomy tree indicates the specific required and optional input parameters. For example: “Source.Volts.DC” would require Volts.

## 6. The SoA Editor Design and Implementation

**Figure 7:**
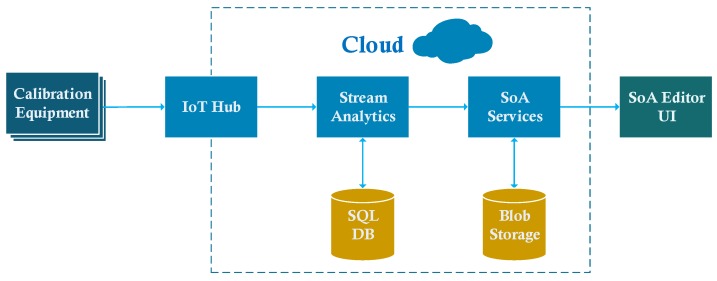
Overall system architecture.

This section provides details of the cloud implementation of the SoA editor. Figure 7 shows the overall architecture of the system. This architecture contains the *core subsystems* defined in the Azure IoT reference architecture [34]. In our solution, calibration equipment setups are IoT devices. During a calibration process, usually, the device under test (DUT) and the reference device are connected to a PC (Personel Computer). This PC can connect to the Internet to send messages to the cloud. This part of the implementation composes of the physical layer of the IoMT architecture.

The IoT Hub is the cloud gateway in IoT applications. It allows scalable and secure two-way communication between IoT devices and the cloud. The data collected by IoT Hub is transferred to another service on the cloud side, such as Stream Analytics or Azure Functions. IoT Hub can tolerate the management of thousands of IoT devices. However, in the SoA editor scenario, scalability is not a critical issue because the number of calibration equipment is not as large as other IoT applications, such as wireless sensor networks. The number of devices may reach thousands when the usage of the application becomes widespread, e.g., comprising all CLs in a country.

**Figure 8:**
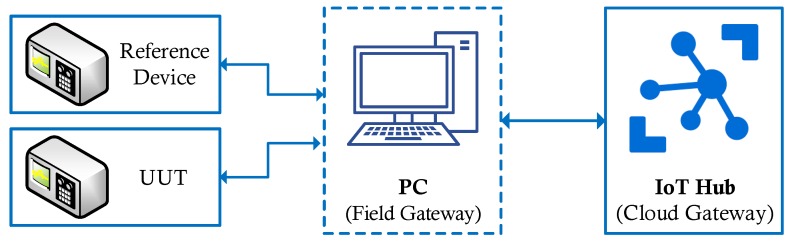
A simple calibration setup connected to the cloud through a field gateway.

In the IoMT architecture, devices in a calibration setup correspond to IoT devices in a typical IoT solution. However, UUT and the reference device do not have to be IP-capable. Generally, they are connected to a PC which is IP-capable and can connect to the cloud. This scenario fits well with the Field Gateway concept presented in [34]. A Field Gateway is used to connect IoT devices to the cloud gateway. This pattern is shown in Figure 8. Because it allows local processing, filtering, or data aggregation, using a field gateway reduces the data transferred to the cloud. In our scenario, the field gateway is a suitable place to do uncertainty calculations. To create an SoA document for a CL, we do not need all measurement data produced in a calibration setup. Uncertainty calculations involve statistical analysis and comparisons against universal standards after collecting a set of calibration data. These calculations can be done locally, and only the resulting value is sent to the cloud to reduce the workload on the cloud side. Our experience with the Metrology.NET platform helps us establishing this kind of setup for automating the calculations in the edge and transferring required data to the cloud.

The job of stream analytics is to pre-process the data collected by IoT Hub. It allows filtering the data based on queries and directing data to other services, such as a database or another cloud service. It also allows for creating user-defined functions to let more complex jobs. As an alternative to the edge computing in the SoA editor scenario, uncertainty calculations to define CMCs in the SoA document can be done in a stream analytics job. Then these values are forwarded to the SoA service. The SoA Service saves SoA data in a database in XML format.

SoA related data and other required databases, UoM Database and Metrology Taxonomy, as shown in Figure 4, are kept as XML files, as MII suggests. The SoA service uses these files to store the data of a CL, adding parameters to the company’s calibration capabilities, etc. Blob Storage is a convenient environment to store these XML files [35], considering the amount of storage needed for the SoA editor case study. For another scenario that large-scale data is collected, Azure Data Lake is the alternative to be used. Azure Data Lake allows storing large amounts of relational and nonrelational data in a distributed manner [34]. It also allows big data analytics that will be beneficial, especially for device producers who want to analyze their device’s calibration performance.

Besides the data directed to the SoA service, a stream analytics job can directly save some amount of data to the storage. For example, all of the calibration data of a specific device may have great importance for its producer. Even though this data is irrelevant for the SoA document itself, it can be significant for other services in the IoMT concept. An SQL database can be employed for this purpose, as shown in Figure 7.

### 6.1. SoA Editor as an Azure Cloud Service

The SoA editor was implemented as a desktop application in [3]. To provide a clear separation between the presentation layer (front-end) and business logic (back-end), the design of the editor follows the Model-View-ViewModel (MVVM) architectural design pattern [36]. The design pattern allows for the concurrent development of the system components. Also, when you need to change the model, there is no need to update the view, and vice versa. The UI (User Interface) of the editor was developed using the Windows Presentation Foundation (WPF) of Microsoft [37].

**Figure 9:**
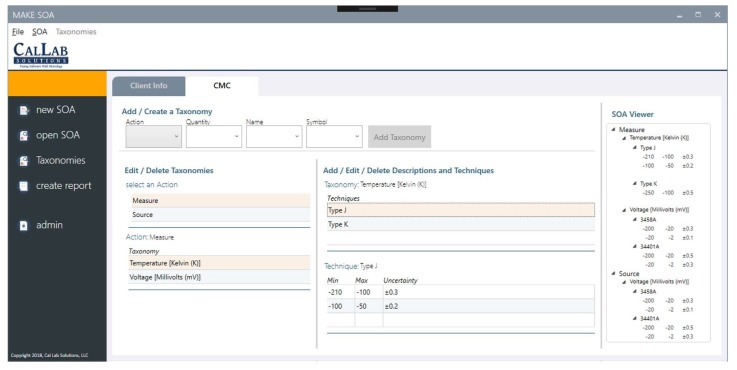
A screenshot from the SoA editor desktop application.

Figure 9 shows a screenshot from the desktop version of the SoA editor. The screen shows different elements that are used in adding a new measurement parameter to a CL scope. Parameters are added based on the predefined measurement taxonomy explained in Section 5.5.

The desktop application is converted to a cloud service to realize the IoMT architecture. Therefore, the editor can work in harmony with other cloud services. The editor’s code run as a service on the cloud is presented in Figure 7 as *SoA Services*. To create the cloud service, the *WPF application* created before is converted to an *Azure Cloud Service* in Microsoft Visual Studio (VS). Both the applications are implemented with C#. The layered implementation of the desktop application enables the conversion to take place easily. The MVVM pattern is converted to ASP.NET Model-View-Controller (MVC) [38] pattern for the cloud application, since it will be used as a web application. Model parts of the applications correspond directly. View-Model and Controller parts require slight changes since the connection methods for the UI elements are different. The View layer for the cloud application is re-written with ASP.NET. The following section provides the key details of the MVC implementation.

### 6.2. Model-View-Controller Pattern

**Figure 10:**
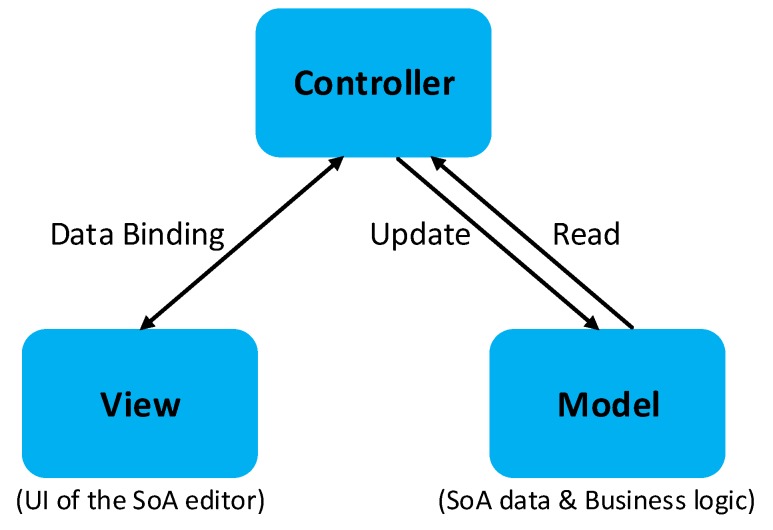
The SoA editor design in MVC.

The SoA editor components based on the MVC pattern are explained in this section. Figure 10 shows the layers. A brief explanation for each layer is provided below.

#### 6.2.1. Model

The Model layer represents the data and business logic of the application. To be decoupled from the Controller, it exposes the data through observables to that layer. The data, as shown in Figure 4, is stored in the MII Cloud Services layer. The Model uses the SoA Schema to handle the read/write of the SoA data, and the Metrology Taxonomy to categorize measurements in a standardized way. The Model also takes advantage of the UoM Database that keeps measurement units in a standard format defined by the MII group.

#### 6.2.2. View

The View layer includes all the elements about the UI of the editor. Its role is to subscribe to the Controller observable(s). The UI events are also passed to the Controller, which are then reflected in the database. The UI provided in Figure 9 is an example of Views. The Controller layer handles the user inputs provided by the UI and does the necessary operations based on the data standards defined in the Model layer.

#### 6.2.3. Controller

The Controller layer behaves as a mediator between the View and the Model layers. It interacts with the Model and supplies observable(s) to the View layer. As an implementation strategy for this layer, the Controller is decoupled from the View, which allows for an interchangeable View component for the editor. It also sends updates made in the View layer back to the Model layer.

### 6.3. Deployment to Azure

**Figure 11:**
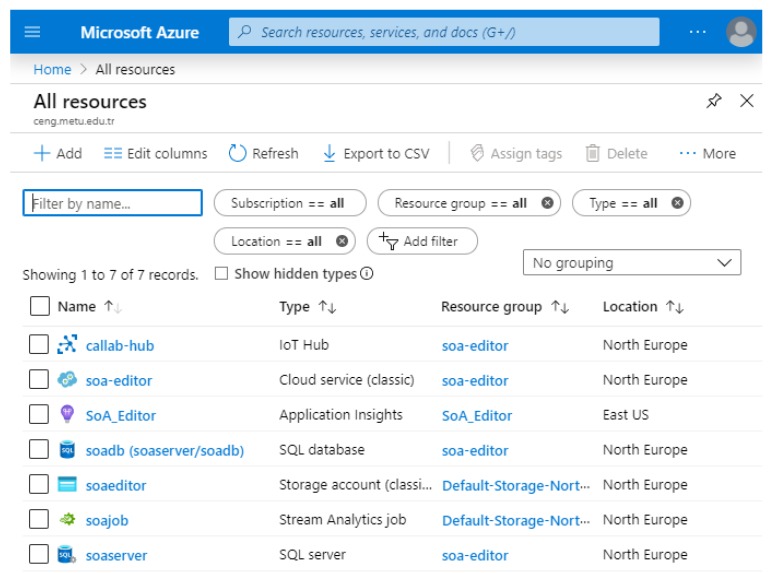
Allocated resources and services on the Azure portal.

The account used for the Azure portal can be the same Microsoft account that is used in VS. In this way, deployment of the locally created service to the Azure can be done easily. The required resources to run the service on cloud (namely, Cloud Service, Storage Account, and Application Insights) can be created simply using a configuration window of VS, and deployment is done in this way. After deployment, Azure provides a link to access the editor’s UI through the created cloud service. Other services that are depicted in Figure 7 (IoT Hub, Stream Analytics, SQL Database, and Blob Storage) are created by Azure Portal, and the required connections are performed among the services to yield a running application. The resources and services used in the SoA editor case study comply with the lowest pricing policy of the Azure. Figure 11 shows the resources created on the Azure portal.

## 7. Implementation Alternatives

This section includes implementation alternatives for the IoMT architecture realization. We briefly explain options for the cloud provider and an alternative application, a test point editor.

### 7.1. Cloud Providers

Although we use Azure in our implementation, other cloud services could be used as well. For example, AWS (Amazon Web Services) [39], Google Cloud [40], and IBM Cloud [41] are other widely used cloud computing platforms.

As an alternative to the Azure application, we explain which AWS services can be used for the SoA editor example. AWS IoT Core is the cloud application that allows communication and management of IoT devices. IoT Core can be used to collect data from CLs and transfer it to the cloud for further processing. AWS IoT Analytics can be used to process collected data, e.g., for filtering or uncertainty calculations in our SoA editor scenario. Amazon Relational Database Service (Amazon RDS) is an option to store calibration data on the cloud. AWS S3 storage can store taxonomies and other XML files that SoA Service uses and produces. SoA service can be implemented on AWS Lightsail that allows creating simple web applications and websites.

### 7.2. The Test Point Editor

IoMT architecture covers various applications and services other than that are used for the SoA editor scenario. A *test point* editor implementation can be another example application for the realization of IoMT, which includes other stakeholders in the industry, namely equipment manufacturers.

Equipment manufacturers provide test points in the manuals that are delivered with the equipment. Calibrators use this device-specific data during the calibration process. These manuals are for humans, and they are not machine-readable. Because there is no standard data format, manufacturers use their way to prepare these manuals. This lack of standardization may cause a vast amount of diversity.

During a manual calibration, CL technicians read and follow calibration manuals. Alternatively, CL can use off-the-shelf software for automation or create its own automation software. For the off-the-shelf software case, CL does not have to deal with test point data as the off-the-shelf software already contains it. When CL uses its own software, it has to translate the human-readable test point data to a machine-readable format. This translation is cumbersome as it requires excessive time and effort.

IoMT architecture may help to handle test point data in a better way. Manufacturers would use a test point editor to create test points. Produced test point data would be stored in the cloud to allow the usage of manufacturers and other stakeholders. Services that would be used by the test point editor are *test point service* and *metrology taxonomy service*. CLs are the consumer of the test points as they use this data during the calibration process. Therefore, CLs would use the test point editor with a different UI or role. It is also possible that other applications in the application layer use the test point service as test points are central to calibration. Calibration automation software might be an example of such applications. Therefore, standardization helps different applications to use the same data as storing and accessing it are handled by a common service. Same as the SoA editor scenario, the physical layer of the test point editor example composed of CL equipment.

## 8. Conclusions and Future Work

We demonstrated the modeling of an SoA editor as a proof of concept towards verification of the IoMT architecture. In previous work, we proposed the IoMT, an IoT-based architectural framework for the calibration industry that is inspired by the MII works. In this work, we present the realization of the framework by implementing the SoA editor as a cloud application. Also, the necessary steps to have a full-scale IIoT scenario by combining physical equipment and cloud services are explained. Microsoft Azure is chosen as the cloud environment of the application. We believe the Metrology community can benefit from the IoMT concept by complying with specific data standards and employing more automation in calibration processes.

SoA encompasses the statement of a CL’s capabilities. It can be of great importance both to CLs and customers who want their equipment calibrated. ABs certify a CL’s capabilities and thus the services offered by them. There were attempts to digitalize the traditional accreditation process. The MII initiative proposes new standards and software tools and components to help digitalize this process. The SoA editor can be classified in this kind of attempt. The traditional accreditation process involves intense data exchange, which is paper-based, thus, cumbersome. The editor facilitates this data exchange among stakeholders. The SoA editor is an open-source project hosted at Github [42]. Therefore, the project is open to contributions from the metrology community.

Bringing automation to the accreditation process also helps with the traceability. Along the process, there may be a need for a high degree of interaction and data exchange between a CL and AB. These may include several rounds of analyses done by an AB on the calibration scope provided by a CL. This also can include some minor and major editions required by an AB to be fulfilled by a CL. Considering all this communication done in an automated manner, the software can keep track of all the steps along the whole process and thus improve the process traceability.

Other than traceability, there are different non-functional concerns to be considered for the IoMT architecture. Some of them are confidentiality, reliability, governance/community process, and usability. Although some of these concerns are handled by the cloud provider, they need to be investigated and addressed appropriately for the architecture to gain acceptance.

Although there are already implemented applications in the application layer of the IoMT architecture and locally in use by some CLs, they are restricted in terms of data sharing with the Metrology community. In the future, these applications should be encouraged to adapt themselves to IoMT architecture and provide service to other stakeholders and CLs. To ensure that more applications are served on IoMT, more common services that are likely to be shared by many applications will be developed and deployed.

## Figures and Tables

**Table 1:** An excerpt from an SoA certificate showing CL scope for two different parameters.

Parameter/Equipment	Range	CMC (±)	Comments
Temperature-Measure	(−50 to 0) °C	0.36 °C	Fluke 744 & T100-250 PRT
	0 °C	0.37 °C	
	(0 to 100) °C	0.41 °C	
	(100 to 250) °C	0.46 °C	
DC Voltage-Measure	(0 to 100) mV	6.8 µV/V + 0.86 µV	HP 3458A
	(0.1 to 1) V	6.0 µV/V + 0.80 µV	
	(1 to 10) V	6.7 µV/V + 1.3 µV	
	(10 to 100) V	7.0 µV/V + 32 µV	
	(100 to 1000) V	7.8 µV/V + 59 µV	

## Abbreviations

The following abbreviations are used in this manuscript:

AB	Accreditation Body
API	Application Programming Interface
AWS	Amazon Web Services
CL	Calibration Laboratories
CMC	Calibration and Measurement Capability
DUT	Device Under Test
GPIB	General Purpose Interface Bus
IaaS	Infrastructure as a Service
IEC	International Electrotechnical Commission
IIC	Industrial Internet Consortium
IIoT	Industrial Internet of Things
IoMT	Internet of Measurement Things
IoT	Internet of Things
ISO	International Organization for Standardization
MathML	Mathematical Markup Language
MII	Metrology Information Infrastructure
MVC	Model-View-Controller
MVVM	Model-View-ViewModel
NCSLI	National Conference of Standards Laboratories - International
OPC-UA	Open Plant Communication Universal Architecture
PaaS	Platform as a Service
PC	Personel Computer
SaaS	Software as a Service
SoA	Scope of Accreditation
SQL	Structured Query Language
UI	User Interface
UML	Unified Modeling Language
UoM	Units of Measure
UUT	Unit Under Test
VS	Visual Studio
WPF	Windows Presentation Foundation
XML	Extensible Markup Language
